# Weekly Calcifediol 100 µg in Women with Vitamin D Deficiency: A *Post Hoc* Analysis of a Randomised Placebo-Controlled Phase II/III Trial

**DOI:** 10.3390/nu18213658

**Published:** 2026-11-04

**Authors:** José Luis Pérez Castrillón, Esteban Jódar-Gimeno, Aintzane García-Bea, Cristina Martínez Ostalé, Inmaculada Gilaberte

**Affiliations:** 1 Department of Internal Medicine, Río Hortega University Hospital, 47012 Valladolid, Spain; uvacastrv@gmail.com; 2 Department of Endocrinology and Nutrition, Quirónsalud Madrid University Hospital, 28223 Madrid, Spain; 3 Department of Medicine, Faculty of Medicine, Health and Sports, Universidad Europea de Madrid, 28670 Villaviciosa de Odón, Spain; 4 Medical Affairs Department, FAES FARMA, 48940 Leioa, Spain; 5 Clinical Development Department, FAES FARMA, 48940 Leioa, Spain

**Keywords:** calcifediol, vitamin D deficiency, 25-hydroxyvitamin D, women, menopause, body mass index

## Abstract

**Background/Objective:** Given the high prevalence of vitamin D deficiency, particularly among older women, this *post hoc* analysis was conducted to evaluate the long-term efficacy and safety of weekly calcifediol 100 µg in women with vitamin D deficiency participating in a randomised, double-blind, phase II/III trial. **Methods:** A total of 261 women were included (calcifediol, *n* = 192; placebo, *n* = 69). The primary objective was to compare the proportions of women achieving serum 25(OH)D concentrations ≥20 ng/mL or ≥30 ng/mL over the 12-month treatment period. Exploratory analyses of body mass index (BMI) and menopausal status were performed. **Results:** Weekly calcifediol 100 µg demonstrated superior efficacy versus placebo from week 4 in women achieving ≥20 ng/mL (68.2% vs. 5.8%; *p* < 0.001) or ≥30 ng/mL (21.4% vs. 0%; *p* < 0.001), and consistently higher proportions were maintained through week 52. Mean 25(OH)D concentrations increased from 12.21 ng/mL at baseline to 39.47 ng/mL at week 16, and remained stable (41–43 ng/mL) thereafter. No significant differences were observed across BMI or menopausal status subgroups. The safety profile of weekly calcifediol 100 µg was similar to placebo, with no evidence of toxicity. **Conclusions:** Weekly calcifediol 100 µg is a safe and effective option for achieving and maintaining adequate vitamin D levels in vitamin D-deficient women over a period of 12 months, and is associated with consistent effects across BMI and menopausal status.

## 1. Introduction

Vitamin D is increasingly being recognised as a pleiotropic factor with endocrine-like activities rather than merely a dietary nutrient. When solar exposure is sufficient, approximately 80% of vitamin D is synthesised in the skin via ultraviolet B (UVB) irradiation, whereas around 20% is obtained from dietary sources [1]. Vitamin D_3_, as the predominant form of vitamin D in the human body, enters circulation and undergoes sequential hydroxylation, first in the liver to 25-hydroxyvitamin D_3_ [25(OH)D_3_, also called calcifediol or calcidiol], the main circulating marker of vitamin D status, and subsequently—primarily in the kidney—to its biologically active form, 1,25-dihydroxyvitamin D_3_ [1,25(OH)_2_D_3_] [1].

Vitamin D status depends strongly on environmental conditions that determine effective UVB exposure. Shorter daylight hours and reduced winter UVB availability are associated with lower serum 25(OH)D concentrations, whereas longer daylight and greater spring–summer sun exposure increase 25(OH)D levels [2,3]. This contributes to substantial seasonal variability in vitamin D status, particularly in regions where winter sunlight is insufficient to support meaningful cutaneous synthesis [2,3]. Lifestyle and cultural factors further compound this seasonal pattern. Indoor working and living habits, reduced outdoor physical activity, and extensive use of photoprotection, including sunscreen and clothing coverage, limit the skin exposure required for endogenous vitamin D synthesis [4,5]. These behaviours help explain why vitamin D deficiency remains highly prevalent even in settings with abundant sunlight, where ambient UVB availability may be offset by limited time outdoors or deliberate sun avoidance [4,5].

Ageing is an important biological factor in this risk. Older adults have reduced cutaneous vitamin D synthesis, less efficient intestinal calcium absorption and age-related changes in hepatic and renal vitamin D metabolism [6]. These factors are particularly relevant in postmenopausal women, in whom low serum 25(OH)D concentrations may coexist with oestrogen deficiency and accelerated bone loss [6,7]. Consequently, postmenopausal women are a clinically important population for assessing and correcting vitamin D deficiency [7].

The strongest medical rationale for correcting low 25(OH)D concentrations remains skeletal health. Vitamin D is central to calcium homeostasis, bone mineralisation and the maintenance of the mineralised skeleton [8]. Deficiency has long been recognised as a major cause of rickets in children and osteomalacia in adults, and it can also contribute to osteoporosis and fracture risk, particularly when calcium balance is inadequate or secondary hyperparathyroidism is present [8].

In addition to its skeletal effects, low vitamin D status has been linked to several chronic conditions, including cardiovascular disease, autoimmune diseases and respiratory infections [1,9]. Low circulating 25(OH)D concentrations in women have been associated with a range of gynaecological and reproductive conditions, including menopausal symptom burden, polycystic ovary syndrome, endometriosis, uterine fibroids and selected adverse pregnancy outcomes. Although these associations should not be taken as proof of causality, they underscore the potential clinical relevance of maintaining adequate vitamin D status across the stages of women’s health [10,11,12,13].

This relevance is reinforced by the high prevalence of vitamin D insufficiency worldwide. In a large pooled analysis of population-based studies, vitamin D deficiency remained highly prevalent both globally and in Europe, with estimates exceeding 50% and higher rates among women than men [14].

Vitamin D supplementation remains the standard approach to restoring adequate 25(OH)D concentrations. Within this context, calcifediol is a fast-acting, potent and well-tolerated option for preventing and correcting vitamin D deficiency [15,16,17]. Owing to its pharmacokinetic profile, calcifediol is suitable for use across the broad spectrum of patients with vitamin D deficiency and may offer particular advantages over cholecalciferol in settings such as obesity, liver disease, malabsorption, or when a rapid increase in 25(OH)D concentrations is clinically desirable [18].

A phase II/III clinical development programme evaluated weekly calcifediol regimens for up to 52 weeks in patients with moderate or severe vitamin D deficiency. Across cohorts, the 100 µg weekly dose resulted in a high percentage of subjects achieving target levels with a favourable safety profile, supporting a subsequent *post hoc* analysis in the overall study population to assess whether this dose provided consistent efficacy and safety across baseline deficiency strata [19,20].

Although the efficacy, safety and pharmacokinetic profile of calcifediol have been described previously in mixed-sex populations and across different dosing regimens, information specifically focused on women remains limited. In particular, little is known about whether clinically relevant female characteristics, such as menopausal status and body mass index, may influence the response to weekly calcifediol supplementation. Given the physiological and hormonal changes associated with menopause, as well as the recognized impact of adiposity on vitamin D metabolism and distribution, exploring the consistency of treatment response across these subgroups may provide clinically relevant information for women’s health.

Building on the previous overall analysis, this *post hoc* analysis was designed to further characterize the response to weekly calcifediol 100 µg versus placebo in women, including exploratory analyses by body mass index (BMI) and menopausal status.

## 2. Materials and Methods

Full eligibility criteria and trial conduct, including treatment procedures, study medication characteristics, administration instructions, follow-up schedules, blood sample collection, and laboratory analyses, have been reported previously [19,20,21].

### 2.1. Participants

**Figure 1:**
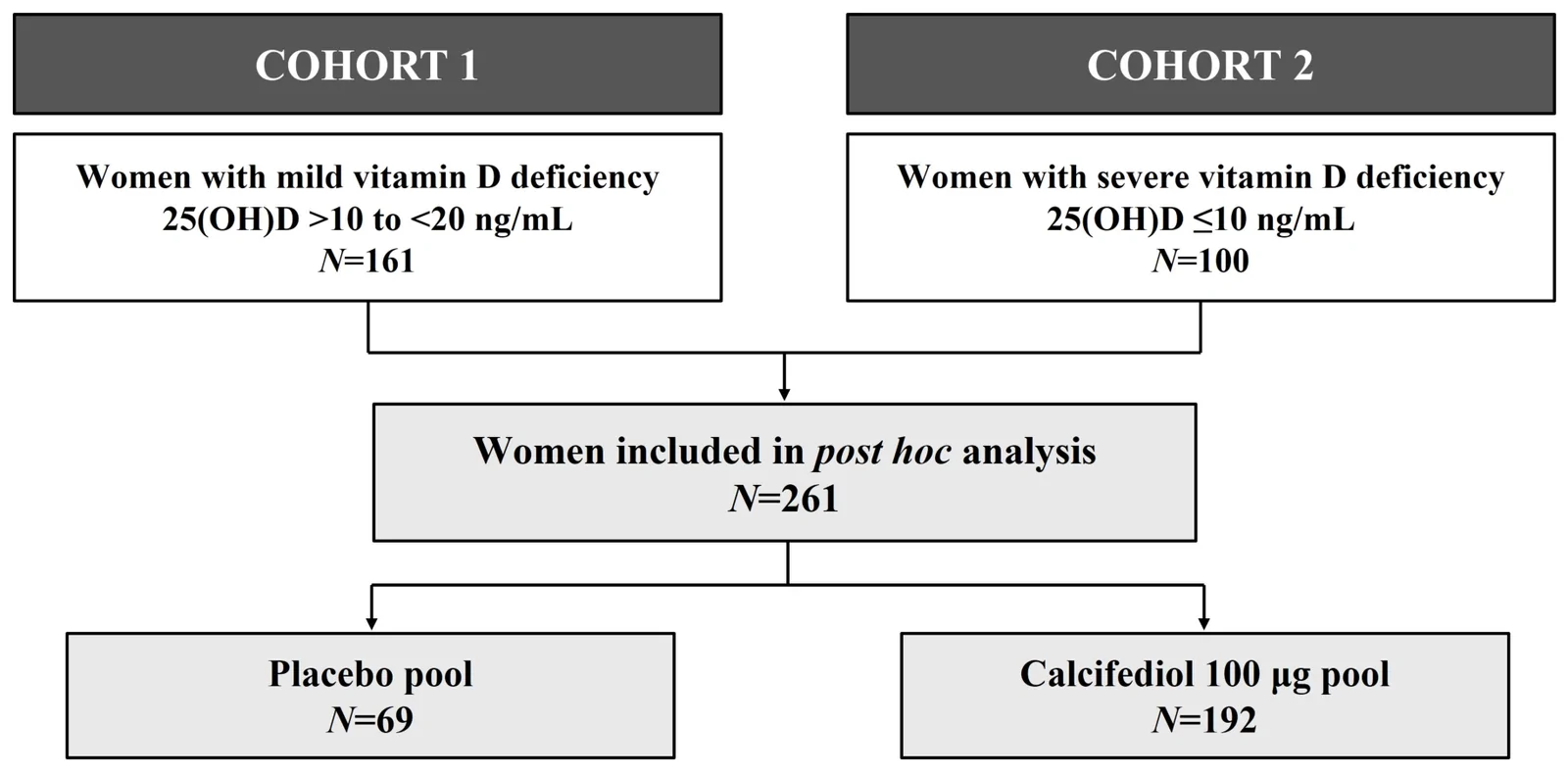
Study population. Women participating in the original study were included in the *post hoc* analysis, as follows: cohort 1, with mild vitamin D deficiency [25(OH)D >10 to <20 ng/mL]; and cohort 2, with severe vitamin D deficiency [25(OH)D ≤10 ng/mL]. Participants were pooled according to treatment allocation to the placebo and calcifediol 100 μg groups.

This women-only *post hoc* analysis included 261 female participants randomised to placebo or once-weekly calcifediol 100 µg soft gelatine capsules who did not receive rescue medication during the trial. Overall, 161 women had mild vitamin D deficiency at baseline, defined as serum 25(OH)D >10 and <20 ng/mL (cohort 1 of the clinical trial), and 100 women had severe vitamin D deficiency at baseline, defined as serum 25(OH)D ≤10 ng/mL (cohort 2 of the clinical trial; Figure 1).

### 2.2. Institutional Review Board

The study was conducted according to the guidelines of the Declaration of Helsinki, and approved by the Institutional Review Board (or Ethics Committee) of Bulgaria: Ethics Committee for Clinical Trials (EKKÈ-4351/16.10.2020; 9 December 20); Czec Republic: Etická komise Fakultní nemocnice v Motole (EK-1271/20; 7 October 2020), Etická komise Nemocnice Pardubického kraje, a.s. (22/01/21), Etická komise Všeobecné fakultní nemocnice v Praze (1819/20 S; 15 October 2020), Etická komise nestátního zdravotnického zarízení Research Site s.r.o. (209128; 5 November 2020); Spain: CEIm Grupo Hospitalario Quirónsalud-Cataluny (25 October 2021); France: Comité de Protection des Personnes Ouest IV—Nantes (87/20_1; 11 March 2021); Italy: Comitato Etico Indipendente presso la Fondazione PTV Policlinico Tor Vergata (180.20 P.U.; 13 October 2020), Comitato Etico Ospedale San Raffaele (307/2020; 11 November 2020), Comitato Etico Università Vanvitelli di Napoli, AOU Vanvitelli—AORN Ospedali dei Colli (276/2020; 23 December 2020), Comitato Etico Milano Area 2 (982_2020; 16 December 2020), Comitato Etico Area vasta Nord Ovest-Sezione autonoma del CE regionale per la Sperimentazione clinica (18257_MAZZANTINI; 7 December 2020), Comitato Etico Lazio Area 1 (17/2001 Rif 6148; 22 January 2021), Comitato Etico Palermo 1—Azienda Ospedaliera Universitaria Policlinico “Paolo Giaccone” (09/2020; 22 October 2020); Serbia: Eti_ cki odbor Srbije (515-20-07537-2020-4515-20-07537-2020-4; 27 May 2021); and Slovakia: Etická komisia Košického samosprávneho kraja (6410/2020/ODDZ-34516; 29 September 2020).

### 2.3. Informed Consent

All subjects involved in the study signed the Informed Consent Form.

### 2.4. Methodology

After screening, participants were independently randomised within each cohort. For the present analysis, only women allocated to the placebo and calcifediol 100 µg arms who did not receive rescue medication were retained, yielding a 1:2 placebo-to-active drug allocation. Overall, 69 women were included in the pooled placebo group and 192 in the pooled calcifediol 100 µg group (Figure 1).

The primary objective of this *post hoc* analysis was to assess and compare the proportions of women achieving serum 25(OH)D concentrations of ≥20 ng/mL or ≥30 ng/mL at weeks 4, 16, 24, 32, and 52.

Secondary objectives included evaluating changes in 25(OH)D concentrations over time, assessing 25(OH)D outcomes by BMI category and menopausal status, and describing the safety profile throughout the 52-week treatment period, with a post-treatment follow-up visit at weeks 55–56.

Weight according to BMI was categorised as normal for values from 18.5 to <25 kg/m^2^, overweight for values from 25 to <30 kg/m^2^, and obesity for values ≥ 30 kg/m^2^. Only 8 participants were classified as underweight (BMI < 18.5 kg/m^2^), so this subgroup was described but not included in comparative analyses.

In the parent trial, the postmenopausal state had been prospectively defined per protocol as the absence of menses for 12 months without an alternative medical cause, and medical and surgical history was recorded in the electronic case report form and coded using MedDRA (version 23.1). For the present post hoc subgroup analysis, menopausal status was therefore derived from these coded medical-history records: postmenopausal women were defined as participants with a MedDRA-coded medical-history term of “menopause” or “postmenopause,” and premenopausal women as those without a menopausal medical-history record and without any of the gynaecological surgical procedures specified below. To avoid potential misclassification, women with medical history terms indicating gynaecological surgery, including “hysterectomy” (12 participants), “salpingectomy” (1 participant) or “hysterosalpingo-oophorectomy” (1 participant) were excluded from the menopausal status subgroup analysis as menopausal status could not be confirmed based on additional information (e.g., laterality of the procedure, hormonal status, or preserved ovarian function).

Safety assessments included treatment-emergent adverse events (TEAEs), ECG recordings, vital signs, and laboratory safety tests, including haematology and biochemistry. The laboratory panel comprised haematology (haemoglobin, haematocrit, red and white blood cell counts with differential, and platelets) and biochemistry (sodium, potassium, glucose, AST, ALT, gamma-glutamyl transferase, creatinine, total protein and albumin), in addition to bone and mineral metabolism parameters (alkaline phosphatase, total serum calcium, phosphorus and PTH). Renal function was monitored by serum creatinine at each laboratory visit, and estimated glomerular filtration rate (eGFR, CKD-EPI) was calculated at screening; severe renal impairment (eGFR < 30 mL/min/1.73 m^2^) was an exclusion criterion. Clinically relevant changes were assessed, and TEAEs were summarised as the percentage of participants in each treatment group experiencing any serious TEAE or any treatment-related TEAE. Changes in bone and mineral metabolism parameters were also analysed and reported as the percentage of participants with abnormal values at baseline and at week 52.

Serum total 25(OH)D concentrations, i.e., the sum of 25(OH)D_2_ and 25(OH)D_3_, were quantified at the central laboratory (LKF, Schwentinental, Germany) using the Elecsys Vitamin D Total II electrochemiluminescence immunoassay (ECLIA) on a cobas^®^ analyser (Roche Diagnostics, Rotkreuz, Switzerland).

### 2.5. Statistical Analysis

All female participants, excluding those who received rescue medication (cholecalciferol) and who received at least one dose of the investigational product, either placebo or calcifediol 100 µg, were included in the *post hoc* safety analyses, forming the safety population. Among these, women with at least one post-baseline assessment of serum 25(OH)D concentration were included in the *post hoc* statistical analyses, forming the full analysis population. Main analyses were performed using SAS^®^ version 9.4 (SAS Institute Inc., Cary, NC, USA) in a validated and secure environment.

Between-group differences in responder rates were assessed using a two-sided test of proportions based on the normal approximation, with significance set at *p* < 0.05. Changes in serum 25(OH)D concentrations over time were analysed using a mixed model for repeated measures (MMRM), with baseline value, visit, treatment, baseline-by-visit interaction and treatment-by-visit interaction as fixed effects. Within-subject correlations were modelled using an unstructured covariance matrix.

Exploratory analyses comparing responder rates across BMI subgroups and across pre or postmenopausal subgroups were conducted in R version 4.5.1 (R Foundation for Statistical Computing, Vienna, Austria). The *prop.test* function was used to perform a two-sided test of proportions based on the normal approximation, with significance set at *p* < 0.05. Analyses were conducted separately at each visit and restricted to participants with non-missing responder status. As a supportive analysis, within-group changes in the proportion of participants with abnormal bone and mineral metabolism values between baseline and week 52 were analysed using McNemar’s exact test on paired data, and between-group comparisons at each visit were analysed using Fisher’s exact test, with Holm–Bonferroni adjustment for multiplicity across the four parameters within each family of tests.

## 3. Results

### 3.1. Demographics and Baseline Characteristics

**Figure 2:**
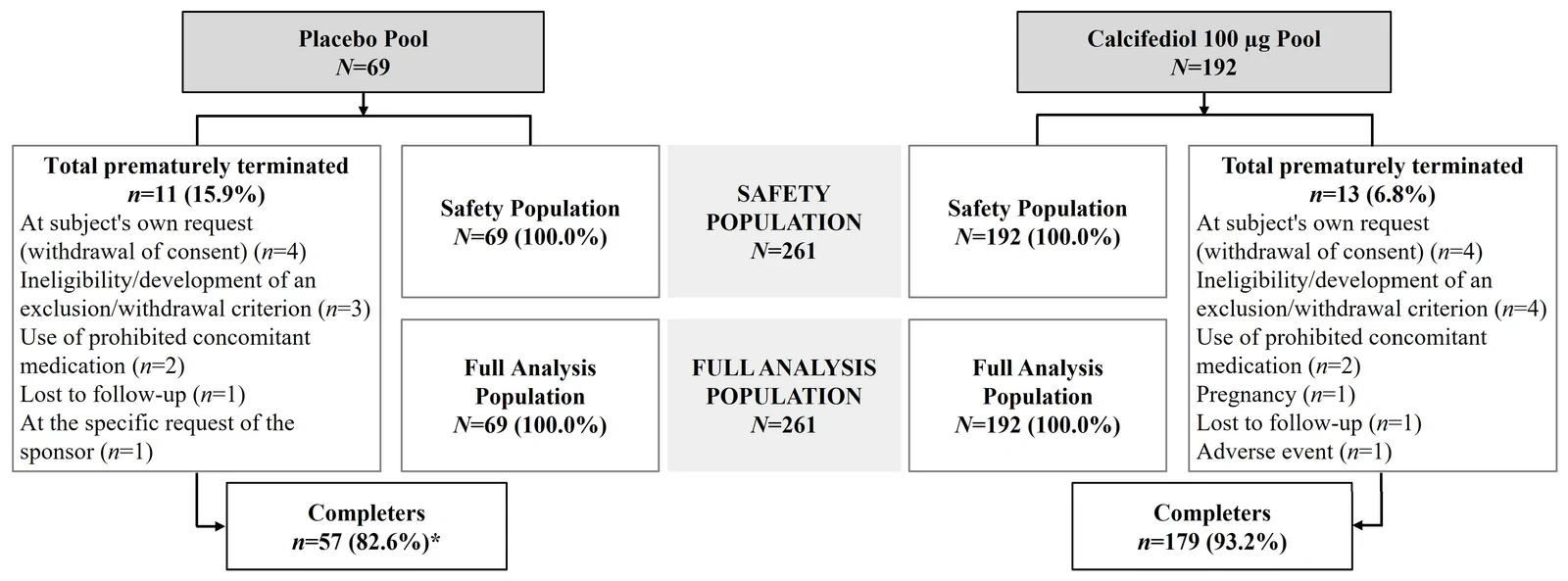
Study flow. The study flow, including individuals in the placebo and calcifediol 100 μg pools, the reasons for premature termination, and the number of subjects included in each study population are shown. * One participant completed treatment but was not included in the last visit analysis due to missing laboratory data.

The safety population comprised 261 women, including 69 in the placebo group and 192 in the calcifediol 100 μg group. All of them followed study procedures at least until the first assessment visit at week 4. Therefore, the full analysis population was identical to the safety population. During the study, 11 women in the placebo group (15.9%) and 13 in the calcifediol 100 μg group (6.8%) prematurely discontinued treatment (Figure 2).

Baseline demographic, anthropometric, and clinical characteristics were generally comparable between treatment groups (Table 1). Mean (SD) age was 49.17 (14.39) years in the placebo group and 52.48 (16.41) years in the calcifediol 100 µg group. At baseline, racial distribution, height, weight, and abdominal circumference were broadly comparable between the placebo and calcifediol groups. Mean BMI was similar between groups (27.66 kg/m^2^ in the placebo group and 28.46 kg/m^2^ in the calcifediol group), although obesity was numerically more frequent among women receiving calcifediol 100 µg than among those receiving placebo (36.13% vs. 23.53%; *p* = 0.08). Menopausal status was also similarly distributed, with postmenopausal women representing 53.13% and 57.30% (*p* = 0.56) of the placebo and calcifediol groups, respectively. Baseline serum 25(OH)D concentrations did not differ significantly between groups, with mean (SD) values of 12.92 (3.56) ng/mL in the placebo group and 12.21 (4.26) ng/mL in the calcifediol 100 µg group (*p* = 0.2123).

Baseline medical conditions were generally comparable between the weekly calcifediol 100 µg and placebo groups (Supplementary Table S1). One of the most frequent conditions was hypertension (26.1% calcifediol vs. 33.9% placebo). Other conditions included dyslipidaemia (13.0% in both groups), hypothyroidism (9.4% calcifediol vs. 11.6% placebo). Moreover, as previously presented, asthma was present at baseline in 7.8% of the calcifediol 100 µg group and 10.1% of the placebo group. Type 2 diabetes mellitus was observed in 9.4% of the calcifediol 100 µg group versus 4.3% of the placebo group.

### 3.2. Proportion of Subjects Achieving Vitamin D Levels ≥20 ng/mL or ≥30 ng/mL over Time

**Figure 3:**
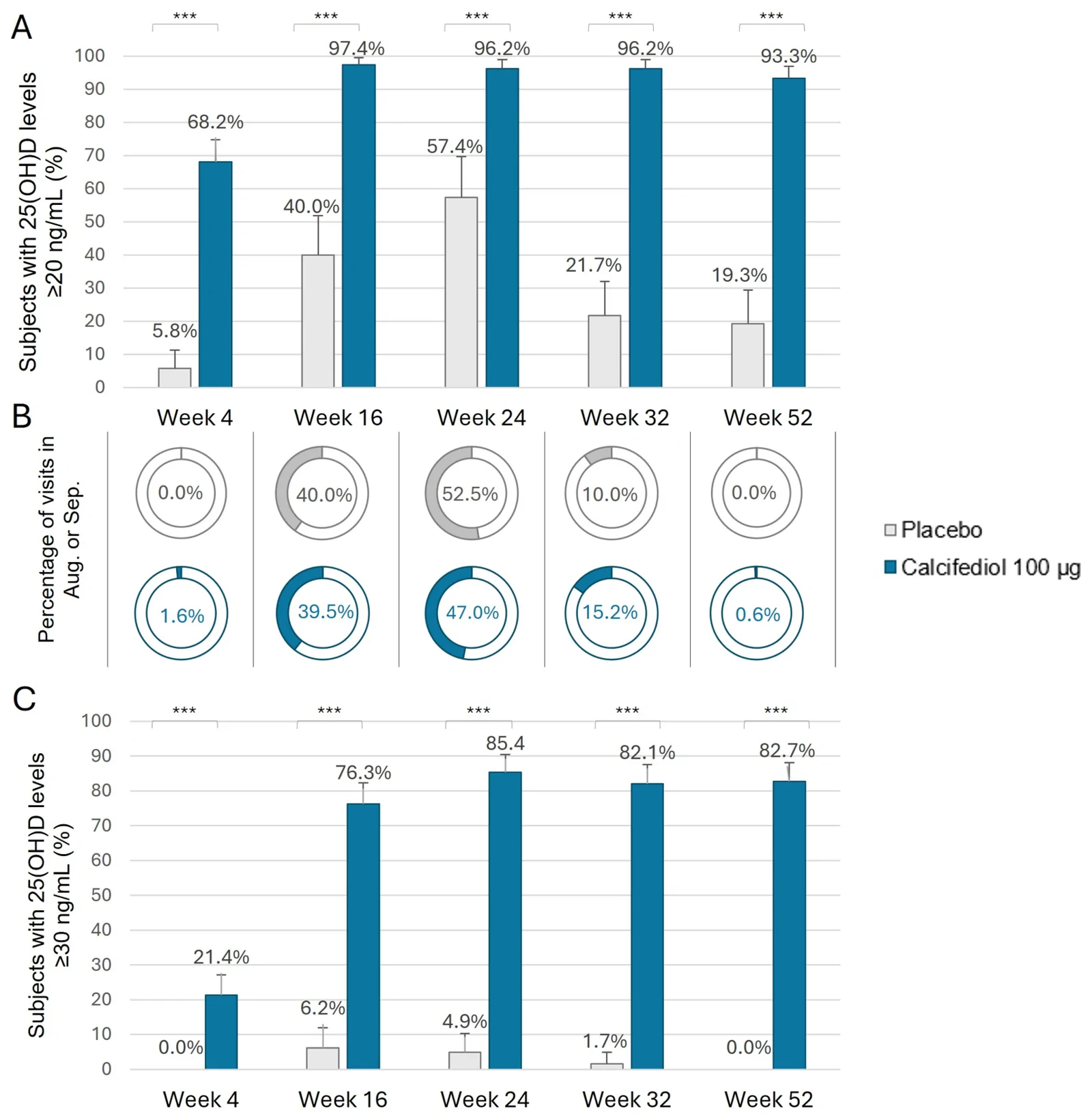
Percentage of participants reaching predefined serum 25(OH)D thresholds and seasonal distribution of visits by treatment group. (**A**) Proportion of participants with serum 25(OH)D levels ≥20 ng/mL at weeks 4, 16, 24, 32, and 52. (**B**) Proportion of participants whose assessment at each timepoint coincided with August (Aug.) or September (Sep.). (**C**) Proportion of participants with serum 25(OH)D levels ≥30 ng/mL at weeks 4, 16, 24, 32, and 52. Error bars indicate 95% confidence intervals for the responder rate. *** *p* < 0.001 for between-group comparisons.

The proportion of women achieving serum 25(OH)D concentrations ≥20 ng/mL was significantly higher in the calcifediol 100 µg group than in the placebo group at all post-baseline visits (*p* < 0.001 for all comparisons; Figure 3A). At week 4, 68.2% of women receiving calcifediol 100 µg had already reached 25(OH)D levels ≥20 ng/mL, compared with 5.8% of those receiving placebo. From week 16 onwards, more than 93% of women in the calcifediol group maintained 25(OH)D concentrations ≥20 ng/mL, whereas the placebo group had lower, fluctuating responder rates that never exceeded 57.4% (week 24).

A similar pattern was observed at the ≥30 ng/mL threshold (Figure 3C). The percentage of women reaching this target was significantly greater with calcifediol 100 µg than with placebo at every post-baseline visit (*p* < 0.001 for all comparisons). At week 4, 21.4% of participants in the calcifediol group achieved 25(OH)D levels ≥30 ng/mL, while none in the placebo group did. From week 16 to week 52, the proportion of responders in the calcifediol group remained consistently above 76%, whereas in the placebo group, the proportion of women reaching ≥30 ng/mL remained low throughout follow-up, peaking at 6.2% at week 16.

The higher placebo responder rates observed at weeks 16 and 24 for both the ≥20 and ≥30 ng/mL thresholds coincided with a greater proportion of visits in August or September (Figure 3B). This seasonal pattern was not observed in the calcifediol group, where responder rates remained high throughout the 52-week period.

### 3.3. 25(OH)D Levels over Time

**Figure 4:**
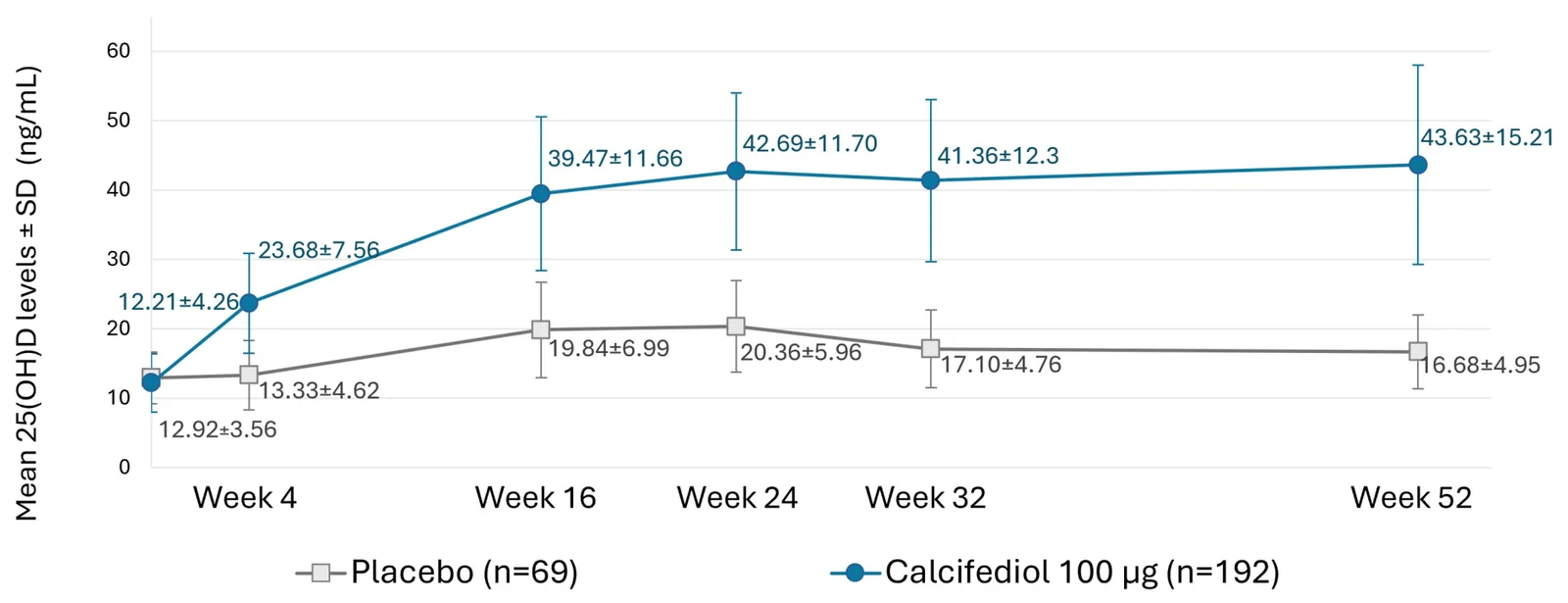
Mean serum 25(OH)D concentrations during follow-up. Mean serum 25(OH)D concentrations measured at baseline and at follow-up visits at weeks 4, 16, 24, 32, and 52 in participants receiving weekly placebo or calcifediol 100 µg. Values are expressed as mean ± SD in ng/mL; error bars indicate SD. According to the MMRM analysis, values were significantly higher at all assessed timepoints following calcifediol treatment versus placebo (*p* < 0.001).

Mean serum 25(OH)D concentrations rose rapidly after starting weekly calcifediol 100 µg and were significantly higher than those in the placebo group at every post-baseline visit (*p* < 0.001; Figure 4). In the calcifediol group, mean (SD) 25(OH)D levels increased from 12.21 (4.26) ng/mL at baseline to 23.68 (7.56) ng/mL at week 4 and 39.47 (11.66) ng/mL at week 16. Thereafter, concentrations remained fairly stable, with mean values above 41 ng/mL.

In the placebo group, mean 25(OH)D concentrations showed only a modest, transient increase, peaking at week 24 at a mean (SD) of 20.36 (5.96) ng/mL, before declining again by week 52 to 16.68 (4.95) ng/mL.

### 3.4. Subgroup Analysis by BMI and Menopausal Status

**Figure 5:**
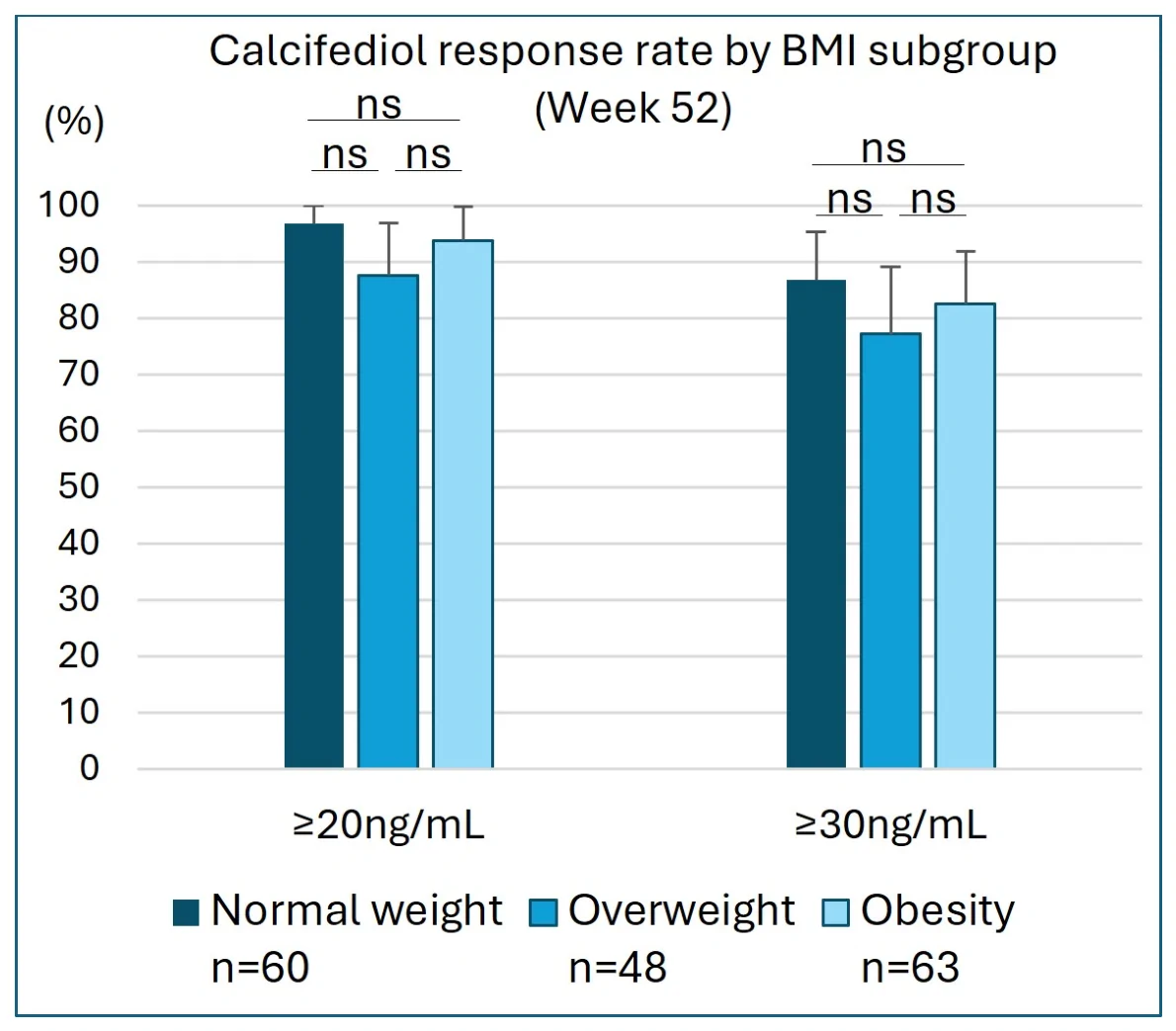
Calcifediol response rate by BMI subgroup at week 52. Response rate (%) among participants receiving calcifediol 100 µg, stratified by BMI subgroup. Bars show the proportion of participants achieving serum 25(OH)D levels ≥20 ng/mL and ≥30 ng/mL at week 52. Error bars indicate 95% confidence intervals for the responder rate. ns, not significant for comparisons between BMI subgroups. Note: Of the 192 participants in the calcifediol 100 µg group, 1 had missing BMI data. Of the remaining 191, the data of 8 underweight participants were not analysed, leaving 183 participants. Of these, 12 discontinued before the visit at week 52, resulting in a total of 171 participants included in this analysis.

Among women receiving calcifediol 100 µg, week-52 responder rates remained comparable across BMI subgroups, with no statistically significant differences at either threshold. Among overweight women, 87.5% (95% CI 78.1–96.9) achieved serum 25(OH)D concentrations ≥20 ng/mL, and 77.1% (95% CI 65.2–89.0) achieved concentrations ≥30 ng/mL. Similarly, 93.7% (95% CI 87.6–99.7) of women with obesity achieved concentrations ≥20 ng/mL, and 82.5% (95% CI 73.2–91.9) achieved concentrations ≥30 ng/mL (Figure 5).

**Figure 6:**
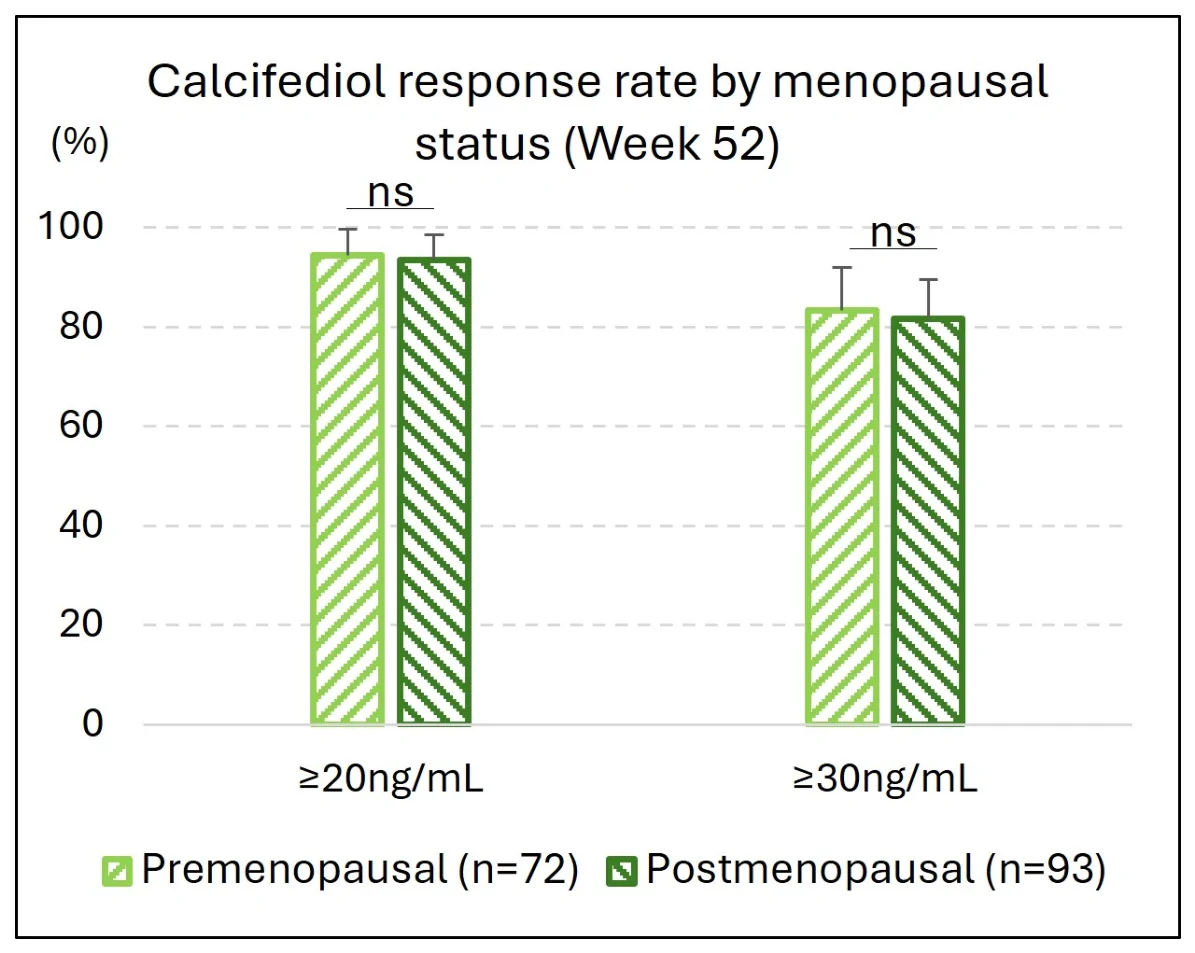
Calcifediol response rate by menopausal status at week 52. Response rate (%) among participants receiving calcifediol 100 µg, stratified by menopausal status. Bars show the percentage of participants achieving serum 25(OH)D levels ≥20 ng/mL and ≥30 ng/mL at week 52. Error bars indicate 95% confidence intervals for the responder rate. ns, not significant for comparisons between menopausal status subgroups. Note: Of the 192 participants in the calcifediol 100 µg group, menopausal status could be classified for 178 (76 premenopausal and 102 postmenopausal; Table 1). Fourteen women with a history of gynaecological surgery for whom menopausal status could not be confirmed were excluded from this analysis. Week-52 rates were calculated among classified participants with a non-missing week-52 assessment.

Responder rates were also consistent when analysed by menopausal status (Figure 6). Baseline serum 25(OH)D concentrations were comparable between premenopausal and postmenopausal women (13.3 and 11.4 ng/mL, respectively), while, as expected, postmenopausal women were older and had a higher mean BMI (Supplementary Table S2). At week 52, premenopausal and postmenopausal women receiving calcifediol 100 µg had similarly high rates of serum 25(OH)D concentrations ≥20 ng/mL (94.4%, 95% CI 89.2–99.7 and 93.5%, 95% CI 88.6–98.5, respectively) and ≥30 ng/mL (83.3%, 95% CI 74.7–91.9 and 81.7%, 95% CI 73.9–89.6, respectively), with no statistically significant differences between groups.

### 3.5. Safety Results

**Figure 7:**
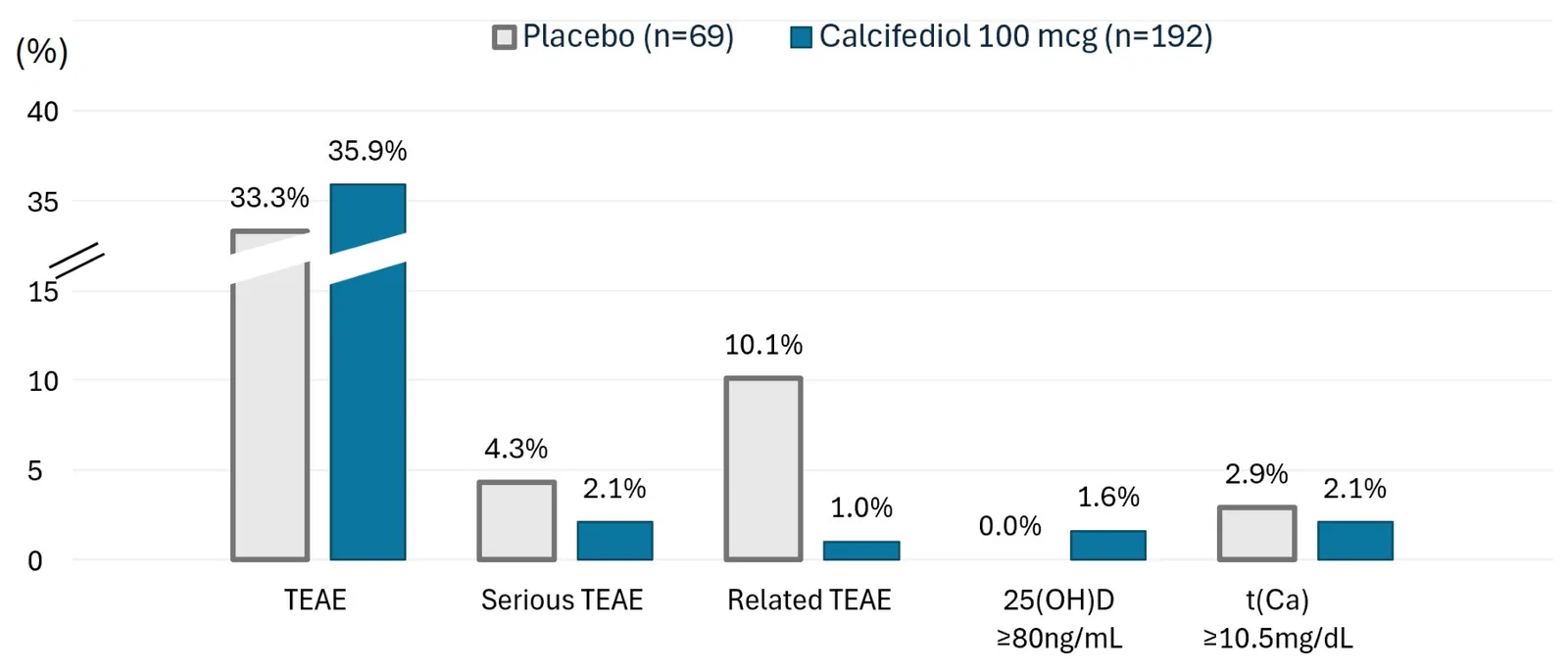
Safety summary in the safety population. Percentage of subjects with any TEAE, serious TEAE, treatment-related TEAE, elevated 25(OH)D levels ≥80 ng/mL, or elevated t(Ca) levels ≥10.5 mg/dL in the placebo and calcifediol 100 μg groups. TEAE, treatment-emergent adverse event; 25(OH)D, 25-hydroxyvitamin D; t(Ca), total serum calcium.

The safety profile of weekly calcifediol 100 µg was similar to placebo throughout the 52-week follow-up period (Figure 7). Overall TEAE rates were comparable between groups, with 33.3% of women receiving placebo and 35.9% receiving calcifediol 100 µg experiencing TEAEs. Treatment-related TEAEs were less frequent in the calcifediol group than in the placebo group (1.0% vs. 10.1%). Most related TEAEs in the placebo group (5/7) were worsening of hypovitaminosis D. Serious TEAEs were reported in 4.3% of participants in the placebo group and 2.1% in the calcifediol group; no TEAEs were considered related to treatment.

Serum 25(OH)D concentrations ≥80 ng/mL were observed only in the calcifediol group, affecting 1.6% of participants; no treatment-related adverse events were reported in these participants. Total serum calcium concentrations ≥10.5 mg/dL occurred at similarly low frequencies in both groups (2.9% with placebo and 2.1% with calcifediol 100 µg; Figure 7). In the calcifediol group, elevated calcium values were not associated with elevated 25(OH)D concentrations.

No clinically relevant changes from baseline and no relevant between-group differences were observed in haematology or biochemistry parameters; mean and median values remained within the reference ranges throughout the study (Tables S3 and S5). Abnormal values reaching ≥5% in either group were comparable between arms, stable over time and largely reflected baseline population characteristics, most frequently elevated glucose, haematocrit and monocyte percentage, while liver-enzyme elevations were infrequent and similar between groups (Tables S4 and S6). Mean serum creatinine remained essentially unchanged in both groups, and the proportion of women with elevated creatinine was low and comparable between arms at baseline and endpoint, with no calcifediol-related renal safety signal (Tables S3 and S4).

Analysis of bone and mineral metabolism parameters showed broadly comparable frequencies of abnormalities in alkaline phosphatase, total serum calcium and phosphorus between treatment groups at baseline and at week 52 (Table 2). The most notable change was a decrease in the proportion of patients with elevated parathyroid hormone levels, from 19.7% at baseline to 4.6% at week 52 in the calcifediol group, compared with a decrease from 22.1% to 17.0% in the placebo group. In a supportive analysis, this within-group reduction reached statistical significance in the calcifediol group (McNemar’s exact test, adjusted *p* < 0.001) but not in the placebo group (adjusted *p* = 1.00), and the between-group difference at week 52 was also significant (Fisher’s exact test, adjusted *p* = 0.046). Notably, the percentage of patients with serum calcium levels >10 mg/dL remained similar at baseline and after 52 weeks treatment with calcifediol 100 µg. No statistically significant within-group changes or between-group differences were observed for alkaline phosphatase, calcium or phosphorus.

## 4. Discussion

In this *post hoc* analysis of women with vitamin D deficiency, weekly calcifediol 100 µg demonstrated superior efficacy compared with placebo, with higher responder rates at both clinically relevant 25(OH)D thresholds (20 and 30 ng/mL) and higher mean serum 25(OH)D concentrations maintained throughout the 52-week treatment period. These differences were statistically significant between groups at all visits and are consistent with findings in the overall population, including both sexes, treated with weekly calcifediol 100 µg [19]. Whereas the efficacy, safety and dose–response of calcifediol have been established previously in the mixed-sex parent population [19,20,21], the present analysis adds evidence specifically in women and, as an exploratory objective, examines whether menopausal status or BMI is associated with the response.

An early rise in serum 25(OH)D levels was observed in the weekly calcifediol 100 µg group, reaching 23.68 ng/mL by week 4, followed by stabilisation at 41–43 ng/mL from week 16 through week 52. This rapid kinetic profile reflects the fact that calcifediol is directly available in the circulation and does not require hepatic hydroxylation, with serum 25(OH)D concentrations peaking approximately 4 h after oral administration [22,23]. The regimen evaluated here was a weekly soft-gel capsule, a formulation whose pharmacokinetic characteristics underlie the rapid onset and the stable, sustained 25(OH)D concentrations observed. These pharmacokinetic properties, as reflected in the present results, support the rapid correction of vitamin D deficiency and the maintenance of stable levels during long-term treatment with calcifediol 100 µg, consistent with previous reports by Jódar-Gimeno et al. [21].

Interestingly, a transient increase in serum 25(OH)D concentrations was also observed in the placebo group during the mid-study period, with responder rates ≥ 20 ng/mL of 40% and 57.4% at weeks 16 and 24, respectively. This pattern is consistent with seasonal sunlight exposure, as a substantial proportion of these visits coincided with August and September, when endogenous vitamin D synthesis is highest, consistent with findings by Karacan et al. [24] and Won et al. [25]. However, this effect was not sustained, as responder rates decreased from week 32 onward, suggesting that sun exposure alone may be insufficient to maintain vitamin D sufficiency over time. This seasonal pattern is clinically relevant because it suggests that apparent improvements in summer should not necessarily prompt treatment discontinuation when the therapeutic goal is to maintain clinically meaningful 25(OH)D concentrations year round. Notably, even at the peak of the summer, responder rates at the ≥30 ng/mL threshold remained very low in the placebo group (maximum 6.2% at week 16), indicating that endogenous vitamin D synthesis alone is insufficient to achieve optimal vitamin D levels, a finding consistent with the recommendations of the Central and Eastern European Expert Consensus Statement on vitamin D [16]. Similarly, Pittaway et al. reported greater seasonal variability in serum 25(OH)D among non-supplemented compared to supplemented individuals [26].

Our exploratory subgroup analysis by BMI showed that weekly calcifediol 100 µg provided consistent efficacy across BMI categories (normal weight, overweight and obesity) throughout the 52-week treatment period. These findings are clinically relevant, as reduced response to vitamin D supplementation with cholecalciferol has been reported in individuals with higher BMI, as described by Tobias et al. [27]. In contrast, no significant differences in response to calcifediol were observed across BMI categories, which may be explained by the linear relationship between dose and serum 25(OH)D concentration [17]. Current clinical guidelines recommend calcifediol as a better supplementation option in patients with obesity [28]. This may be particularly relevant for postmenopausal women and women with polycystic ovary syndrome (PCOS), in whom overweight and obesity are highly prevalent [29,30,31].

Similarly, comparable rates of women achieved 25(OH)D ≥20 ng/mL or ≥30 ng/mL at week 52 in the premenopausal and postmenopausal groups receiving calcifediol 100 µg, with no statistically significant differences between groups. These findings are consistent with those reported by Occhiuto et al., who showed that calcifediol supplementation maintained stable 25(OH)D levels over a 2-year period in postmenopausal women with vitamin D insufficiency [32]. Importantly, vitamin D deficiency can be observed throughout a woman’s lifespan and may be influenced by hormonal fluctuations, changes in body composition and reduced sun exposure [33], affecting both premenopausal and postmenopausal women [34] (prevalence rates of up to 80% in European postmenopausal populations) [11]. In this context, weekly calcifediol 100 µg may represent a practical dosing strategy across different life stages, particularly in routine gynaecological practice, where a single weekly regimen could simplify treatment decisions across different ages and clinical contexts.

Although the analysis was not conceived as a prevention study, the sustained maintenance of 25(OH)D concentrations above clinically relevant thresholds during the 52-week treatment period suggests that weekly calcifediol 100 µg may also help prevent recurrence of vitamin D insufficiency after repletion. In addition, considering the mean increase of approximately 30 ng/mL from baseline and the linear dose–response curve [17], weekly calcifediol 100 µg could be considered as a supplementation strategy in patients at risk of vitamin D deficiency (e.g., obesity or malabsorption), given the wide toxicity threshold margin (generally considered >100 ng/mL) [35]. Additionally, no clinically relevant differences in safety were observed between the weekly calcifediol 100 µg and placebo groups. These observations are in line with the favourable safety profile reported previously in which calcifediol 100 µg showed balanced TEAEs rates and no evidence of toxicity over 52 weeks [21], and a similar safety profile observed across both mild and severe vitamin D deficiency cohorts [19]. Furthermore, the clinically relevant reduction in the proportion of patients with secondary hyperparathyroidism that was observed in the calcifediol group compared with placebo suggests a metabolic benefit.

Taken together, the findings of this *post hoc* study support the use of weekly calcifediol 100 µg as a safe and predictable long-term strategy in women with vitamin D deficiency, combining rapid achievement of target 25(OH)D concentrations with sustained maintenance over time despite seasonal variability in endogenous vitamin D synthesis. The safety results observed during the 52 weeks also support the suitability of long-term use in clinical practice.

This analysis has some limitations. It was restricted to women, was exploratory in nature and was not specifically powered for subgroup comparisons. Although treatment effects were explored within the different subgroups, no formal treatment-by-BMI or treatment-by-menopausal-status interaction analyses were performed. Therefore, the findings should not be interpreted as evidence of differential treatment responses between subgroups, but rather as supportive evidence of a consistent response to weekly calcifediol across the female populations evaluated. As an inherent limitation of the *post hoc* design, menopausal status was classified retrospectively from recorded medical history rather than from prospectively collected reproductive data. In addition, dietary vitamin D intake was neither assessed nor controlled (the parent protocol imposed no restrictions with respect to food) and, together with seasonal sun exposure, may have contributed to the temporal variability in serum 25(OH)D, particularly in the placebo group. In addition, the exclusion of participants who received rescue medication may have introduced selection bias, primarily affecting the placebo group and potentially enriching it with spontaneous responders, leading to a more conservative estimate of calcifediol efficacy. However, key strengths include its derivation from a well-designed phase II/III trial with a year-long treatment and centralised assessment of 25(OH)D, both of which support the methodological robustness of the efficacy and safety findings in women. Moreover, this study includes exploratory subgroup analyses by BMI and menopausal status, enhancing the clinical relevance of our results. Finally, because the present analysis was restricted to women, it could not address whether sex modifies the relationship between BMI and the response to calcifediol; studies prospectively designed and adequately powered to test a sex × BMI interaction would be a valuable next step to determine whether the consistency observed here extends to differences between male and female populations.

## 5. Conclusions

In conclusion, weekly calcifediol 100 µg, administered as a weekly soft-gel capsule, was safe and effective in women with vitamin D deficiency over a 52-week treatment period. Efficacy was consistent across BMI categories, seasons, and menopausal status in these exploratory subgroup analyses. These findings suggest that weekly calcifediol 100 µg may represent a safe and practical dosing strategy for the treatment and prevention of vitamin D deficiency across different ages and clinical settings.

## Figures and Tables

**Table 1:** Baseline demographic, anthropometric and menopausal characteristics by treatment group.

		Placebo (*n* = 69)	Calcifediol 100 µg (*n* = 192)
Race	Black	0 (0%)	1 (0.52%)
	White	67 (97.10%)	186 (96.88%)
	Asian	0 (0%)	1 (0.52%)
	Other *	2 (2.90%)	4 (2.08%)
Age (years)	Mean	49.17	52.48
	SD	14.39	16.41
	Min	22.00	18.00
	Max	75.00	84.00
Height (cm)	Mean	164.01	163.36
	SD	6.52	6.85
	Min	152.00	142.00
	Max	182.00	180.00
Weight (kg)	Mean	74.29	75.83
	SD	18.9	18.98
	Min	46.00	41.60
	Max	149.20	146.00
Abdominal circumference	Mean	90.64	92.74
	SD	16.45	17.58
	Min	66.00	54.00
	Max	130.00	138.00
BMI (kg/m^2^)	Mean	27.66	28.46
	SD	7.04	7.11
	Min	18.8	17.40
	Max	49.3	54.10
BMI subgroup	Underweight	0 (0%)	8 (4.19%)
	Normal weight	31 (45.59%)	62 (32.46%)
	Overweight	21 (30.88%)	52 (27.23%)
	Obesity	16 (23.53%)	69 (36.13%)
Menopause group	Premenopausal	30 (46.88%)	76 (42.70%)
	Postmenopausal	34 (53.13%)	102 (57.30%)

* Other includes Arabic, Hispanic, Hispano-Latino, Moroccan, Maghrebi and multiracial or multiethnic categories. Max, maximum; min, minimum; SD, standard deviation.

**Table 2:** Incidence of bone and mineral metabolism abnormalities at baseline and week 52. The table presents the total number of participants included in the safety population (*N*), the number with evaluable data for each parameter (*n*), and the proportion of participants (%) with values below or above the predefined reference range.

Parameter		Placebo (*N* = 69)		Calcifediol 100 µg (*N* = 192)	
		Baseline	Week 52	Baseline	Week 52
Alkaline phosphatase	*n*	69	57	191	179
	Low (%)	1.4	3.5	2.1	2.8
	High (%)	7.2	3.5	5.8	3.4
Total serum calcium	*n*	69	57	191	179
	Low (%)	1.4	0.0	1.0	1.1
	High (%)	0.0	1.8	5.8	5.6
Phosphorus	*n*	69	57	191	179
	Low (%)	0	5.3	2.6	0.6
	High (%)	4.3	1.8	0.5	2.2
Parathyroid hormone	*n*	68	53	188	174
	Low (%)	0	0	0	0
	High (%)	22.1	17.0	19.7	4.6

Normal reference ranges: alkaline phosphatase 40–129 U/L; total serum calcium 8.6–10 mg/dL; phosphorus 0.81–1.45 mmol/L; parathyroid hormone 15–65 pg/mL.

## Data Availability

Data access requests to clinical_rd@faes.es will be possible for five years following article publication.

## Supplementary Materials

The following supporting information can be downloaded at: https://www.mdpi.com/article/10.3390/nu18213658/s1, Table S1: Comparison of ongoing medical conditions between treatment arms; Table S2: Summary of Mean Changes from Baseline in Biochemistry Parameters at Visit 4 and Visit 7 (Safety Set); Table S3: Summary of Mean Changes from Baseline in Biochemistry Parameters at Visit 4 and Visit 7 (Safety Set); Table S4: Incidence of Abnormal Biochemistry Parameters Reported in ≥5.0% of Subjects at Baseline or Endpoint (Safety Set); Table S5: Summary of Mean Changes from Baseline in Haematology Parameters at Visit 4 and Visit 7 (Safety Set); Table S6: Incidence of Abnormal Haematology Parameters Reported in ≥5.0% of Subjects at Baseline or Endpoint (Safety Set).
