# Supplementary material for: Weekly Calcifediol 100 µg in Women with Vitamin D Deficiency: A Post Hoc Analysis of a Randomised Placebo-Controlled Phase II/III Trial
Source: Nutrients. 2026 Nov 4;18(21):3658. doi: 10.3390/nu18213658 (PMC13765258; doi:10.3390/nu18213658)
Supplement: Supplementary file 1 [file nutrients-18-03658-s001.zip › nutrients-4599861-supplementary.pdf]

Supplementary Materials

Weekly Calcifediol 100 µg in Women with Vitamin D Deficiency: A *Post Hoc* Analysis of a Randomised Placebo-Controlled Phase II/III Trial

Table S1. Comparison of ongoing medical conditions between treatment arms

**Baseline condition (SOC) (PT)**	**Placebo (*n* = 69)**	**%**	**Calcifediol 100 µg (*n* = 192)**	**%**	**Total**
Blood and lymphatic system disorders					
Anaemia	4	5.8%	4	2.1%	8
Endocrine disorders					
Hypothyroidism	8	11.6%	18	9.4%	26
Hepatobiliary disorders					
Hepatic steatosis	4	5.8%	7	3.6%	11
Immune system disorders					
Seasonal allergy	5	7.2%	10	5.2%	15
Metabolism and nutrition disorders					
Obesity	17	24.6%	69	35.9%	86
Dyslipidaemia	9	13.0%	25	13.0%	34
Type 2 diabetes mellitus	3	4.3%	18	9.4%	21
Diabetes mellitus	3	4.3%	11	5.7%	14
Hypercholesterolaemia	3	4.3%	11	5.7%	14
Musculoskeletal and connective tissue disorders					
Back pain	6	8.7%	11	5.7%	17
Osteoarthritis	2	2.9%	11	5.7%	13
Spinal pain	4	5.8%	6	3.1%	10
Psychiatric disorders					
Insomnia	4	5.8%	3	1.6%	7
Respiratory, thoracic and mediastinal disorders					
Asthma	7	10.1%	15	7.8%	22
Rhinitis allergic	3	4.3%	16	8.3%	19
Surgical and medical procedures					
Hysterectomy	4	5.8%	15	7.8%	19
Vascular disorders					
Hypertension	18	26.1%	65	33.9%	83
Peripheral venous disease	4	5.8%	10	5.2%	14

Table S2. Summary of Mean Changes from Baseline in Biochemistry Parameters at Visit 4 and Visit 7 (Safety Set)

	**Premenopausal (*n*=76)**	**Postmenopausal (*n*=102)**	**Total classified (*n*=178)**
**Age (years)**			
Mean (SD)	36.5 (10.7)	64.3 (8.3)	52.4 (16.7)
Range (min–max)	18.0–57.0	49.0–84.0	18.0–84.0
**Race, n (%)**			
White	71 (93.4)	101 (99.0)	172 (96.6)
Asian	1 (1.3)	0 (0.0)	1 (0.6)
Black or African American	1 (1.3)	0 (0.0)	1 (0.6)
Other	3 (3.9)	1 (1.0)	4 (2.2)
**Anthropometry, mean (SD)**			
Height (cm)	164.7 (6.2)	162.3 (7.3)	163.3 (6.9)
Weight (kg)	71.6 (18.6)	78.0 (19.1)	75.3 (19.1)
Body mass index (kg/m^2)	26.4 (6.5)	29.7 (7.3)	28.3 (7.2)
Abdominal circumference (cm)	85.7 (15.8)	97.3 (17.6)	92.3 (17.8)
**Baseline serum 25(OH)D (ng/mL)**			
Mean (SD)	13.3 (4.2)	11.4 (4.1)	12.2 (4.2)
Range (min–max)	4.7–19.8	4.9–20.0	4.7–20.0
**BMI category, n (%)**			
Underweight (<18.5)	7 (9.2)	1 (1.0)	8 (4.5)
Normal (18.5 to <25)	27 (35.5)	33 (32.4)	60 (33.7)
Overweight (25 to <30)	25 (32.9)	22 (21.6)	47 (26.4)
Obesity (≥30)	17 (22.4)	45 (44.1)	62 (34.8)
Not available	0 (0.0)	1 (1.0)	1 (0.6)

Data are shown for the 178 women whose menopausal status could be classified (76 pre-menopausal and 102 post-menopausal). A further 14 women could not be assigned to either category because of a medical-history record of gynaecological surgery (12 hysterectomy, 1 salpingectomy and 1 hysterosalpingo-oophorectomy) that precluded confirmation of menopausal status. One post-menopausal woman had no recorded weight, body mass index or abdominal circumference and is shown under “Not available” for BMI category; continuous anthropometric summaries are based on participants with available data. SD, standard deviation; BMI, body mass index; 25(OH)D, 25-hydroxyvitamin D.

Table S3. Summary of Mean Changes from Baseline in Biochemistry Parameters at Visit 4 and Visit 7 (Safety Set)

Parameter	Visit	Placebo (N = 69)		Calcifediol 100 µg (N = 192)	
		Mean Change from Baseline			
		n	Mean (SD)	n	Mean (SD)
Sodium (mmol/L)	Visit 4	65	-0.2 (2.27)	189	0.1 (2.46)
	Visit 7	57	0.8 (2.14)	179	0.7 (2.26)
Potassium (mmol/L)	Visit 4	65	-0.06 (0.652)	188	0.07 (0.367)
	Visit 7	57	-0.23 (0.741)	178	-0.01 (0.440)
AST (GOT) (U/L)	Visit 4	64	-1.7 (7.79)	189	-0.8 (14.66)
	Visit 7	56	1.3 (11.98)	179	-1.8 (13.83)
ALT (GPT) (U/L)	Visit 4	64	-0.7 (13.19)	189	-0.4 (17.92)
	Visit 7	56	2.0 (15.14)	179	-0.7 (16.77)
Gamma-GT (U/L)	Visit 4	65	-1.0 (15.97)	189	-2.7 (24.75)
	Visit 7	57	-0.3 (11.02)	179	-1.5 (34.09)
Creatinine (mg/dL)	Visit 4	65	-0.007 (0.099)	189	0.016 (0.110)
	Visit 7	57	-0.009 (0.083)	179	0.015 (0.085)
Albumin (g/L)	Visit 4	65	0.3 (2.54)	189	0.0 (2.73)
	Visit 7	57	0.8 (2.50)	179	1.0 (2.77)
Total protein (g/L)	Visit 4	65	0.3 (2.83)	189	0.2 (3.83)
	Visit 7	57	1.2 (3.31)	179	1.0 (3.96)
Glucose (mg/dL)	Visit 4	65	3.7 (19.59)	189	1.0 (19.19)
	Visit 7	57	2.1 (16.61)	179	3.0 (29.91)

N = participants in the safety set per group; n = participants with data available; SD = standard deviation.

Table S4. Incidence of Abnormal Biochemistry Parameters Reported in ≥5.0% of Subjects at Baseline or Endpoint (Safety Set)

**Parameter**	**Result**	**Placebo (N = 69)**				**Calcifediol 100 μg (N = 192)**			
		**Baseline**	**n**	**Endpoint**	**n**	**Baseline**	**n**	**Endpoint**	**n**
AST (GOT) (U/L)	n	68		68		192		191	
	High	3 (4.4)		2 (2.9)		10 (5.2)		8 (4.2)	
ALT (GPT) (U/L)	n	68		68		192		191	
	High	3 (4.4)		5 (7.4)		14 (7.3)		12 (6.3)	
Gamma-GT (U/L)	n	69		68		192		191	
	High	4 (5.8)		5 (7.4)		18 (9.4)		19 (9.9)	
Creatinine (mg/dL)	n	69		68		192		191	
	High	6 (8.7)		6 (8.8)		14 (7.3)		16 (8.4)	
Glucose (mg/dL)	n	69		68		192		191	
	Low	4 (5.8)		2 (2.9)		5 (2.6)		6 (3.1)	
	High	14 (20.3)		13 (19.1)		39 (20.3)		46 (24.1)	

N = participants in the safety set per group; n = participants with data available at the visit; % is based on n at each visit. Low/High defined by the central-laboratory reference range. Endpoint = last non-missing post-baseline value (Visit 7/EDV).

Table S5. Summary of Mean Changes from Baseline in Haematology Parameters at Visit 4 and Visit 7 (Safety Set)

Parameter	Visit	Placebo (N = 69)		Calcifediol 100 µg (N = 192)	
		Mean Change from Baseline			
		n	Mean (SD)	n	Mean (SD)
Haemoglobin (g/dL)	Visit 4	65	-0.09 (0.575)	186	-0.07 (0.740)
	Visit 7	57	-0.18 (0.733)	178	-0.01 (0.828)
Haematocrit (%)	Visit 4	65	1.42 (2.451)	186	1.52 (2.814)
	Visit 7	57	-0.71 (2.432)	178	-0.04 (2.570)
Red blood cell count (10^{12}/L)	Visit 4	65	-0.028 (0.216)	185	-0.035 (0.262)
	Visit 7	57	-0.036 (0.242)	177	-0.011 (0.274)
White blood cell count (10^{9}/L)	Visit 4	65	-0.04 (1.334)	186	0.06 (1.664)
	Visit 7	56	0.19 (1.811)	177	0.11 (1.809)
Neutrophils (%)	Visit 4	64	-0.02 (6.177)	185	-0.90 (8.628)
	Visit 7	56	-0.72 (6.881)	177	-1.07 (8.313)
Neutrophils abs. (10^{9}/L)	Visit 4	64	-0.042 (1.056)	185	-0.038 (1.440)
	Visit 7	56	0.028 (1.398)	177	-0.004 (1.522)
Eosinophils (%)	Visit 4	64	0.35 (1.499)	185	0.32 (2.082)
	Visit 7	56	0.19 (1.333)	177	0.18 (1.976)
Eosinophils abs. (10^{9}/L)	Visit 4	64	0.019 (0.100)	185	0.026 (0.145)
	Visit 7	56	0.023 (0.119)	177	0.016 (0.136)
Lymphocytes (%)	Visit 4	64	0.03 (5.376)	185	0.55 (7.582)
	Visit 7	56	0.57 (5.742)	177	0.76 (7.578)
Lymphocytes abs. (10^{9}/L)	Visit 4	64	0.005 (0.487)	185	0.052 (0.506)
	Visit 7	56	0.134 (0.550)	177	0.085 (0.551)
Monocytes (%)	Visit 4	64	-0.36 (2.199)	185	0.02 (1.744)
	Visit 7	56	-0.02 (1.835)	177	0.14 (1.894)
Monocytes abs. (10^{9}/L)	Visit 4	64	-0.037 (0.150)	185	0.004 (0.149)
	Visit 7	56	0.008 (0.094)	177	0.014 (0.163)
Basophils (%)	Visit 4	64	0.00 (0.227)	185	0.02 (0.328)
	Visit 7	56	-0.03 (0.274)	177	-0.01 (0.391)
Basophils abs. (10^{9}/L)	Visit 4	64	-0.000 (0.021)	184	0.002 (0.021)

	Visit 7	56	-0.001 (0.020)	176	0.000 (0.025)
Platelet count (10^9/L)	Visit 4	64	2.6 (36.35)	186	-5.4 (48.30)
	Visit 7	56	6.5 (38.98)	178	1.9 (49.14)

N = participants in the safety set per group; n = participants with data available; SD = standard deviation.

Table S6. Incidence of Abnormal Haematology Parameters Reported in ≥5.0% of Subjects at Baseline or Endpoint (Safety Set)

**Parameter**	**Result**	**Placebo (N = 69)**		**Calcifediol 100 μg (N = 192)**	
		**Baseline n**	**Endpoint n**	**Baseline n**	**Endpoint n**
Haemoglobin (g/dL)	n (data available)	69	68	191	191
	Low	4 (5.8)	5 (7.4)	19 (9.9)	13 (6.8)
Haematocrit (%)	n (data available)	69	68	191	191
	High	13 (18.8)	10 (14.7)	42 (22.0)	42 (22.0)
Red blood cell count (10^{12}/L)	n (data available)	69	68	190	191
	High	5 (7.2)	4 (5.9)	19 (10.0)	14 (7.3)
White blood cell count	n (data available)	69	68	191	191
	High	6 (8.7)	8 (11.8)	18 (9.4)	13 (6.8)
Eosinophils (%)	n (data available)	69	68	190	191
	High	2 (2.9)	4 (5.9)	11 (5.8)	10 (5.2)
Lymphocytes (%)	n (data available)	69	68	190	191
	Low	4 (5.8)	3 (4.4)	10 (5.3)	12 (6.3)
	High	5 (7.2)	4 (5.9)	8 (4.2)	14 (7.3)
Monocytes (%)	n (data available)	69	68	190	191
	High	17 (24.6)	14 (20.6)	34 (17.9)	36 (18.8)
Platelet count (10^9/L)	n (data available)	68	68	191	191
	High	2 (2.9)	7 (10.3)	14 (7.3)	18 (9.4)

N = participants in the safety set per group; n = participants with data available at the visit; % is based on n at each visit. Low/High defined by the central-laboratory reference range. Endpoint = last non-missing post-baseline value (Visit 7/EDV).
